# Intimate partner violence during pregnancy and its association with preterm birth and low birth weight in Tanzania: A prospective cohort study

**DOI:** 10.1371/journal.pone.0172540

**Published:** 2017-02-24

**Authors:** Geofrey Nimrod Sigalla, Declare Mushi, Dan Wolf Meyrowitsch, Rachel Manongi, Jane Januarius Rogathi, Tine Gammeltoft, Vibeke Rasch

**Affiliations:** 1 Institute of Public Health, Kilimanjaro Christian Medical University College, Moshi, Tanzania; 2 Department of Health, Evangelical Lutheran Church in Tanzania, Arusha, Tanzania; 3 Department of Public Health, University of Copenhagen, Copenhagen, Denmark; 4 Department of Anthropology, University of Copenhagen, Copenhagen, Denmark; 5 Department of Obstetrics and Gynecology, Odense University Hospital, Odense, Denmark; 6 Department of Clinical Research, University of Southern Denmark, Odense, Denmark; Stellenbosch University, SOUTH AFRICA

## Abstract

**Introduction:**

Intimate partner violence (IPV) is a public health problem that affects millions of women worldwide. The role of violence as an underlying factor in poor birth outcomes remains an area where strong evidence is lacking. The aim of this study was to determine the association between intimate partner violence (IPV) and preterm delivery (PTB) and low birth weight (LBW).

**Materials and methods:**

A prospective cohort study was conducted among 1112 pregnant women attending antenatal care in Moshi–Tanzania. The women were enrolled before 24 weeks gestation, followed-up at week 34 to determine exposure to violence during pregnancy, and after delivery to estimate gestation age at delivery and birth weight. Logistic regression analysis was performed to assess the association between exposure to IPV during pregnancy and PTB and LBW while adjusting for possible confounders. In addition, stratified analysis based on previous history of adverse pregnancy outcome was performed.

**Results:**

One-third of the women experienced IPV during pregnancy, 22.3% reported emotional, 15.4% sexual and 6.3% physical violence. Women exposed to physical IPV were three times more likely to experience PTB (AOR = 2.9; CI 95%: 1.3–6.5) and LBW (AOR = 3.2; CI 95%: 1.3–7.7). Women with previous adverse pregnancy outcomes and exposure to physical IPV had a further increased risk of PTB (AOR = 4.5; CI 95%: 1.5–13.7) and LBW (AOR = 4.8; CI 95%: 1.6–14.8) compared to those without previous history of adverse outcome.

**Conclusion:**

Women who are exposed to IPV during pregnancy are at increased risk of PTB and LBW. The risk is even stronger if the women additionally have suffered a previous adverse pregnancy outcome. Interventions addressing IPV are urgently needed to prevent occurrence and reoccurrence of PTB and LBW.

## Introduction

Intimate partner violence (IPV), defined as “a behavior by an intimate partner or ex-partner that causes physical, sexual or psychological harm”, is a public health problem that affects more than one-third of all women worldwide [1,2]. The prevalence of IPV during pregnancy in Africa ranges from 2% to 57% [3]. In Tanzania, the prevalence of IPV is 41–56% [4,5] and 27% of pregnant women have experienced physical and/or sexual violence[6] while the prevalence of physical violence during pregnancy is 7–12% [4,7].

Globally, an estimated 12.9 million children are born before 37 gestational weeks, which translates to 9.6% of all children being born preterm [8]. Over three-quarters of all preterm births (PTB) occur in Africa and Asia. In addition, 15.5% of all children are born with a low birth weight (LBW), defined as a birth weight below 2,500 grams, and 95.6% of these children are born in low-income countries [9]. PTB and LBW have devastating effects on the child’s health and are the leading causes of death in children under 5 years [10]. Therefore, attempts to determine factors that are associates with PTB and LBW in low-income countries are needed to contribute towards reduction of morbidity and mortality of children.

Violence during pregnancy is associated with depression and anxiety, inadequate prenatal care, poor maternal weight gain, unhealthy lifestyle (smoking, alcohol consumption, and poor diet) [6,7], and HIV/AIDS [11,12]. Some studies have reported an association between IPV and PTB and/or LBW [13–15] whereas other studies have reported no association [16,17]. The conflicting findings may arise from an underreporting of IPV exposure and consequently a misclassification of IPV cases, which yield falsely insignificant associations. The majority of these studies have not used longitudinal study designs, which are needed to document causal inference. Very little is known about the risks of IPV and recurrence of PTB and LBW.

Antenatal care programs acknowledge the effect maternal health has on the child’s development; however, the role of violence as an underlying factor in women’s ill-health during pregnancy and in poor birth outcomes, remains an area where strong evidence is lacking. For most low-income countries where IPV, PTB and LBW are prevalent, a thorough description of the causal association between IPV and PTB and LBW is lacking.

Considering the public health importance of IPV and the fact that PTB and LBW are important risk factors for infant mortality in sub-Saharan Africa, the present study aimed to determine the association between IPV exposure during pregnancy and PTB and LBW in Moshi Municipality, northern Tanzania. The study additionally examined how IPV was associated with PTB and LBW among women who have suffered a previous adverse pregnancy outcome.

## Materials and methods

A prospective cohort study was performed among pregnant women attending antenatal care in Moshi Municipality, Tanzania. The women were recruited from two primary level health facilities and followed after delivery. The study lasted from March 2014 to May 2015 and inclusion criteria were being pregnant with a gestational age below 24 weeks and willingness to be followed for the entire study period. Women with multiple pregnancies and women who were planning to deliver outside Moshi Municipality were excluded from the study.

Data on socio-demographic and reproductive health characteristics were collected at the time of recruitment (S1 File). During a follow-up interview at 34 weeks gestation, the women’s exposure to emotional, sexual, and physical violence was determined (S2 File). Within 48 hours after delivery, documentation of gestational age and measurement of birth weight was done. The duration of the first and second interviews was 45 to 60 minutes; the post-delivery interview lasted for 5–10 minutes. Those women who had delivered before 34 weeks of gestation were interviewed within 2 weeks after delivery to assess their exposure to IPV.

### Participants and data collection procedure

In all, 1,133 out of 4,250 pregnant women who attended antenatal care fulfilled the inclusion criteria and 1,123 women accepted to participate and were interviewed. Seven women (0.6%) did not turn up for the 34 weeks follow-up interview and four women (0.4%) experienced a pregnancy loss before 24 weeks gestation. Of the 1,112 enrolled women, 35 gave birth before the 34^th^ gestational week and 1,077 gave birth between 34 and 44 gestational weeks.

When registered at the antenatal clinic, participants underwent screening by a nurse midwife and were thereafter invited to participate in the study. Ultrasound measurements of crown-rump length (CRL) for gestational age below 13 weeks and head circumference (HC) for gestational age 13–24 weeks [18] were used to determine the gestational age. Each participant was allocated a unique identification number. Information on socioeconomic and reproductive health characteristics was obtained through a face-to-face interview questionnaire in *Swahili*. Interviews at delivery were conducted at the health facility (if the woman had delivered at a health facility) or at home (in the case of a home delivery). All interviews were conducted in privacy. However, children younger than two years who accompanied the women were allowed to be present during the interview. The research assistants were trained mature female nurses (aged 40 years and above) with experience in maternal and child health research.

### Measures

#### Experience of IPV

The Swahili version of the “WHO Multi-Country Study on Women Health and Domestic Violence against Women” tool, which previously has been used in Tanzania [5], was adopted for this study. In the present study, the assessment period of “past 12 months” in the original tool was changed to “during this pregnancy”. To measure physical violence, the women were asked six questions, namely if, during the index pregnancy, their partner had slapped, pushed, hit, kicked, choked them, or threatened to use or actually used an object that could hurt them. Acts that defined emotional violence were inquired after using four questions; being insulted, humiliated, intimidated, or threatened by the partner. Exposure to sexual violence was assessed by three questions where the women were asked if they had been physically forced to have sexual intercourse, had sexual intercourse without freely giving consent, or been forced to perform a humiliating or degrading sexual act. Experience of any physical, sexual, or emotional violence was considered if a woman reported at least one act of violence from her partner during the index pregnancy.

#### Birth outcomes

Gestational age at delivery was calculated based on the ultrasound examination performed at inclusion. PTB was defined as a delivery before 37 completed weeks of gestation. For the comparison group, deliveries after 37 completed weeks of gestation were defined as births at term. Newborns were weighed immediately after delivery or within 48 hours after delivery for women who delivered at home. LBW was defined as a birth weight of less than 2,500 grams and the comparison group, normal birth weight, defined as birth weight of 2,500 grams or more.

#### Socioeconomic and reproductive health characteristics

At baseline, information on age, education level, and occupation as well as status of cohabitation (living together or apart) was gathered.

#### Reproductive health characteristics

Data on parity, number of children, and if the pregnancy was planned were collected. We assessed previous history of adverse pregnancy outcomes defined as whether the women had previously experienced any pregnancies that ended in miscarriage, stillbirth, PTB, and/or LBW. Tobacco use during pregnancy (yes or no) and consumption of alcohol during pregnancy (yes or no) were determined at enrollment where any consumption of tobacco and/or alcohol classified the response as a ‘yes’.

#### Biological measures and morbidity assessment

At enrollment, blood pressure, weight, and height were measured and the body mass index (BMI) was calculated. During follow-up interviews, information on the women’s blood pressure, weight, HIV status, and hemoglobin level was retrieved from their antenatal card. A participant’s history of diabetes mellitus and hypertension was assessed during the first interview.

### Statistical analysis

Responses obtained from participants were double entered in EpiData version 2.0.3.15, and exported to the Statistical Package for Social Studies (SPSS) program version 20.0 for analysis (S3 File and S4 File). To measure whether the proportional distribution of any IPV differed between different women’s background characteristics, Pearson chi-square test for categorical variables or Fisher’s exact test were used appropriately.

In preparation for bivariate and multivariable logistic regression, outcome variables were coded as dichotomous: birth weight (1 = below 2,500 grams and 0 = ≥ 2,500 grams) and gestation at delivery (1 = below 37 weeks gestation and 0 = ≥ 37 weeks gestation). To determine the association between exposure to IPV during pregnancy, previous adverse pregnancy outcomes and PTB and LBW, crude odds ratio (OR) with 95% confidence intervals (CI) were calculated by bivariate logistic regression. The influence of physical violence was estimated through multivariable logistic regression while including all factors that were significant in the crude analysis (model 1). A second multivariable logistic regression analysis was performed where physical violence plus other covariates that could influence PTB and/or LBW such as age, educational level, occupation, and alcohol consumption during pregnancy were included (model 2). Finally, since history of previous adverse pregnancy outcome was considered potentially to influence birth outcomes, a third multivariable logistic regression analysis was performed where previous PTB and previous LBW were added (model 3). To identify effect modification, stratified analyses of the association between physical violence and PTB and LBW respectively were performed on the basis of previous history of adverse pregnancy outcomes.

### Ethical considerations

Ethical approval of the study was obtained from the Kilimanjaro Christian Medical University College Research and Ethical Review Committee (RERC) with certification number 592 of 2014 with extension in 2015. All participants who met the inclusion criteria were informed of the full nature of study and signed informed consent to participate in the study. The WHO ethical and safety recommendations for researching domestic violence against women were followed [19]. Meetings with stakeholders supporting women who experience violence were conducted and based on their input, support service material, including legal assistance, police support, and maternal and child health services were developed. The materials were given to all participants regardless of whether they reported violence or not. If the woman had signs of impending violence and needed help, contact was made with relevant support institutions after the woman’s consent had been obtained.

## Results

Most women (98.8%) delivered at a health facility, and 95% delivered vaginally. The age of participants ranged from 18 to 44 years, with a mean age of 26 years (Standard deviation, SD = 5.8 years). Most participants (79%) were aged 20 to 35 years, had completed primary education only (61%), were self-employed as farmers or in their own business (46%), were living with their partners (90%), and had one to two children (48%). One in ten (11%) participants consumed alcohol during pregnancy, less than 1% smoked, 53% had anemia, and 2% were underweight (Table 1).

**Table 1:** Characteristics of the study participants, and the prevalence of exposure to at least one type of violence during pregnancy (n = 1112, if no other indication).

	Total (% of total)	Among women with IPV^1^ (%)	Among women with no IPV (%)	p–value^2^
*Age (years)*				
18–20	143 (12.9)	39 (27.3)	104 (72.7)	
21–35	881 (79.2)	271 (30.8)	610 (69.2)	0.70
>35	88 (7.9)	27 (30.7)	61(69.3)	
*Level of completed education*				
Primary	674 (60.6)	207 (30.7)	467 (69.3)	
Secondary	389 (35.0)	114 (29.3)	275 (70.7)	0.77
College	49 (4.4)	16 (32.7)	33 (67.3)	
*Occupation*				
Employed	178 (16.0)	56 (31.5)	122 (68.5)	
Self employed^3^	511 (46.0)	175 (34.2)	336 (65.8)	0.01
Unemployed	423 (38.0)	106 (25.1)	317 (74.9)	
*Smoking*				
Yes	3 (0.3)	0 (0.0)	3 (100.0)	0.56
No	1109 (99.7)	337 (30.4)	772 (69.9)	
*Any alcohol intake during pregnancy*				
Yes	123 (11.1)	56 (45.5)	67 (54.5)	<0.01
No	989 (88.9)	281 (28.4)	708 (71.6)	
*High blood pressure during pregnancy*				
Yes	47 (4.2)	9 (19.1)	38 (80.9)	0.12
No	1065 (95.8)	328 (30.8)	737 (69.2)	
*Anemia during pregnancy (n = 866)*				
<11.0 g/dl	456 (52.7)	145 (31.8)	311 (68.2)	0.33
≥ 11.0 g/dl	410 (47.3)	117 (28.5)	293 (71.5)	
*Body mass index*				
<18.5	24 (2.2)	8 (33.3)	16 (66.7)	0.95
≥18.5	1088(97.8)	329 (30.2)	759 (69.8)	
*Parity*				
0	426 (38.3)	115 (26.9)	313 (73.1)	
1–2	532 (47.8	167 (31.3)	367 (68.7)	0.12
3–6	154 (13.8)	55 (36.7)	95 (63.3)	

^1^ Physical, emotional, and/or sexual violence.

^2^ Results of chi-square test that show statistical difference of exposure to violence.

^3^Self employed include farmers and small business.

### Prevalence of IPV

Overall, 30.3% reported having experienced at least one type of violence when assessed at 34^th^ week of their pregnancy. Emotional violence was the most common form of violence (22.8%) followed by sexual violence (15.4%), and physical violence (6.3%). Exposure to at least one type of violence was significantly more likely to occur among women who were self-employed and who consumed alcohol during pregnancy (Tables 1 and S1).

### Association between IPV and PTB

The mean gestational age at delivery was 276 days (SD = 14 days). Of all deliveries, 88 (7.9%) were preterm (< 37 complete weeks). The associations between exposure to IPV and PTB are presented in Table 2. Women who reported exposure to physical violence during pregnancy had a three times increased risk of PTB when compared to women who were not exposed to physical violence during pregnancy (crude OR = 2.9; 95% CI 1.5–5.6). In contrast, exposure to emotional or sexual violence was not found to be significantly associated with increased risk of PTB. After adjusting for previous history of miscarriage and preterm birth, women who were exposed to physical violence had a 2.7 times increased risk of PTB (Model 1: AOR = 2.7; 95% CI 1.2–5.9). When further adjustment was done to control for the influence of the women’s age, education level, occupation, and alcohol consumption, exposure to physical violence was found to be associated with a 2.8 times increased risk of PTB (Model 2: AOR = 2.8; 95% CI 1.3–6.5). In the final model, where additional adjustment was performed to control for the influence of previous PTB and previous LBW, women who experienced physical violence exerted by their partner during pregnancy were found to be three times more likely to give birth preterm when compared to women who were not exposed to physical violence (Model 3: AOR = 2.9; CI 95%: 1.3–6.5).

**Table 2:** Association between preterm birth with IPV during pregnancy and previous adverse pregnancy outcomes (n = 1112, if no other indication).

Study characteristic	Total (%)	Among women with IPV (Prevalence in %)	Among women with no IPV (Prevalence in %)	Crude OR (95%CI)	Model 1* AOR (95%CI)	Model 2** AOR (95%CI)	Model 3*** AOR (95%CI)
*Physical Violence*							
Yes	70 (6.3)	13 (18.6)	57(81.4)	2.9 (1.5–5.6)	2.7 (1.2–5.9)	2.8 (1.3–6.5)	2.9 (1.3–6.5)
No	1042 (93.7)	75 (7.2)	967(92.8)	1.0			
*Emotional Violence*							
Yes	254 (22.8)	20 (7.9)	234(92.1)	1.0 (0.6–1.7)	-		-
No	858 (77.2)	68 (7.9)	790(92.1)	1.0			
*Sexual Violence*							
Yes	171 (15.4)	17 (9.9)	154(90.1)	1.4 (0.8–2.4)	-		-
No	941 (84.6)	71 (7.5)	870(92.5)	1.0			
*Previous miscarriage (n = 686)*							
Yes	130 (19.0)	20 (15.4)	110(84.6)	2.6 (1.5–4.7)	2.3 (1.3–4.3)	2.3 (1.3–4.3)	2.2 (1.1–4.1)
No	556 (81.0)	36 (6.5)	523(93.6)	1.0			
*Previous preterm birth (n = 679)*							
Yes	22 (3.2)	6 (27.3)	16(72.7)	4.7 (1.7–12.4)	3.2 (1.1–9.1)	3.3 (1.1–9.6)	3.8 (0.9–16.6)
No	657 (96.8)	49 (7.5)	611(92.6)	1.0			
*Previous LBW (n = 676)*							
Yes	33 (4.9)	5 (15.2)	28(84.8)	2.2 (0.8–5.9)			
No	643 (95.1)	49 (7.6)	597(92.4)	1.0			

* Include all factors which were significant in the results of crude analysis:—physical violence, previous miscarriage and previous preterm birth.

** Adjusted for age, education level, occupation, and alcohol intake during pregnancy.

*** Adjusted for age, education level, occupation, alcohol intake during pregnancy, history of previous preterm birth, and history of previous low birth weight.

### Association between IPV and LBW

The mean birth weight was 3,133 grams (SD = 520 grams) and ranged between 500 and 5,000 grams. A total of 74 newborns (6.7%) had a birth weight below 2,500 grams. The results of the analysis of the association between exposure to IPV and LBW are presented in Table 3. Women who reported exposure to physical violence during pregnancy had a close to four times increased risk of LBW when compared to women who were not exposed to physical violence during pregnancy (crude OR = 3.7; 95% CI 1.9–7.1) whereas exposure to emotional or sexual violence were not associated with an increased risk of LBW. After adjusting for previous history of miscarriage, preterm birth, and low birth weight, exposure to physical violence was associated with a three times increased risk of LBW (Model 1: AOR = 3.1; 95% CI 1.3–7.0). When further adjustment was done to control for the effect of the women’s age, education level, occupation, and alcohol consumption during pregnancy, exposure to physical violence was associated with a 3.4 times increased risk of LBW (Model 2: AOR = 3.4; 95% CI 1.4–7.9). Finally, when history of previous PTB and LBW were entered in the model, the strength of the association decreased and women who were exposed to physical violence then had an increased risk of 3.2 times of delivering a LBW child (Model 3: AOR = 3.2; 95% CI: 1.3–7.7). In addition, a significant association was found between any history of miscarriage in previous pregnancies and giving birth to a LBW child. Hence, women with physical violence experience and a previous experience of miscarriage were 2.4 times more likely to deliver a LBW child in comparison with women who had not experienced a miscarriage (Model 3: AOR = 2.4; 95% CI: 1.2–4.6).

**Table 3:** Associations between low birth weight with IPV during pregnancy and previous adverse pregnancy outcomes (n = 1112, if no other indication).

Study characteristic	Total (%)	Among women with IPV (Prevalence in %)	Among women with no IPV (Prevalence in %)	Crude OR (95%CI)	Model 1* AOR (95%CI)	Model 2** AOR (95%CI)	Model 3*** AOR (95%CI)
*Physical Violence*							
Yes	70 (6.3)	13 (18.6)	57(81.4)	3.7 (1.9–7.1)	3.1 (1.3–7.0)	3.4 (1.4–7.9)	3.2 (1.3–7.7)
No	1042 (93.7)	61 (5.9)	981(94.1)	1.0	1.0		
*Emotional Violence*							
Yes	254 (22.8)	19 (7.5)	235(92.5)	1.2 (0.7–2.0)	-		-
No	858 (77.2)	55 (6.4)	803(93.6)	1.0			
*Sexual Violence*							
Yes	171 (15.4)	17 (9.9)	154(90.1)	1.7 (1.0–3.0)	-		-
No	941 (84.6)	57 (6.1)	884(85.2)	1.0			
*Previous miscarriage (n = 686)*							
Yes	130 (19.0)	18 (13.8)	112(86.2)	3.0 (1.6–5.7)	2.4 (1.3–4.8)	2.8 (1.5–5.2)	2.4 (1.2–4.6)
No	556 (81.0)	28 (5.0)	531(95.0)	1.0			
*Previous preterm birth (n = 679)*							
Yes	22 (3.2)	6 (27.3)	16(72.7)	5.9 (2.2–16.1)	2.9 (0.7–12.0)		
No	657 (96.8)	39 (5.9)	621(94.1)	1.0			
*Previous LBW (n = 676)*							
Yes	33 (4.9)	6 (18.2)	27(81.8)	3.5 (1.4–9.1)	1.8 (0.5–6.8)		
No	643 (95.1)	38 (5.9)	608(94.1)	1.0			

* Include all factors which were significant in the results of crude analysis:—physical violence, previous miscarriage, previous preterm birth and previous LBW.

** Adjusted for age, education level, occupation, and alcohol intake during pregnancy.

*** Adjusted for age, education level, occupation, alcohol intake during pregnancy, history of previous preterm birth, and history of previous low birth weight.

As indicated in Table 4, the prevalence of physical violence during pregnancy was higher among women who had experienced a previous adverse pregnancy outcome (10.4%) compared to those without such an experience (6.9%). Women with a previous history of miscarriage, stillbirth, PTB, or LBW who had been physically abused during pregnancy had a significantly increased risk of PTB (OR 4.5, 95% CI 1.5–13.7) and LBW (OR 4.8, 95% CI 1.6–14.8) compared to women who had not been exposed to physical violence. In the group of women without previous adverse pregnancy outcome, the associations were less strong and insignificant (OR 2.1, 95% CI 0.7–6.2 and OR 2.1, 95% CI 0.6–7.5, respectively).

**Table 4:** Association of physical violence and LBW and PTB stratified by history of previous adverse obstetric history (n = 672).

WITHOUT previous adverse obstetric history (n = 508)		Total n (%)	Yes n (%)	No n (%)	Crude OR (95%CI)
*Preterm birth*					
Physical Violence	Yes	35 (6.9)	4 (11.4)	31 (88.6)	2.1 (0.7–6.2)
	No	473 (93.1)	28 (5.9)	445 (94.1)	1.0
*Low birth weight*					
Physical Violence	Yes	35 (6.9)	3 (8.6)	32 (91.4)	2.1 (0.6–7.5)
	No	473 (93.1)	20 (4.2)	453 (95.8)	1.0
**WITH** previous adverse obstetric history (n = 164)*					
*Preterm birth*					
Physical Violence	Yes	17 (10.4)	6 (35.3)	11 (64.7)	**4.5 (1.5–13.7)**
	No	147 (89.6)	16 (10.9)	131 (89.1)	1.0
*Low birth weight*					
Physical Violence	Yes	17 (10.4)	6 (35.3)	11 (64.7)	**4.8 (1.6–14.8)**
	No	147 (89.6)	15 (10.2)	132 (89.8)	1.0

*Women with any history of stillbirth, miscarriage, low birth weight, or preterm birth.

## Discussion

According to our knowledge, this study is one of very few cohort studies in low-income countries that has assessed the association between IPV and PTB and/or LBW. The results indicate that women who are exposed to violence during pregnancy are at significantly increased risk for giving birth preterm and/or having a low birth weight child. Women with previous adverse pregnancy outcome have an even higher risk of both IPV and recurrent PTB and LBW.

IPV during pregnancy was reported by 30% of the women, a finding which is in line with an earlier Tanzanian study where 27% of pregnant women were physically or sexually abused during pregnancy [6]. More than 6% of the women were exposed to physical violence. In other Tanzanian studies, physical violence during pregnancy has been reported to be 7–12% [4,7].

In this study, 6.7% of all infants delivered were of LBW, a finding that is lower than the UNICEF and WHO estimates of 12% for Eastern and Southern Africa but in congruence with previously reported Tanzanian prevalence rates of 6.6–9.4% [20,21]. Based on ultrasound assessment of gestational age, we found the prevalence of PTB to be 7.9%. Comparable prevalence rates (5.3%) have been reported in a cohort of Tanzanian pregnant women who were enrolled before 24 weeks gestation (confirmed by ultrasound scan) [22]. However, a systematic analysis of preterm births in 184 countries showed that 11.1% of all deliveries were PTB with a mean rate of 12.3% in sub-Saharan African countries [10]. The reported differences in prevalence rate of PTB may reflect that pregnant women are exposed to different living conditions, which may be associated with increased or decreased risk of PTB. Conversely, the estimation of gestational age in low income countries most often relies on last normal menstrual period or on self-reported gestational age, and concern thus prevail about how accurately gestational age is determined [13,16]. This may be especially pertinent for women who book late for antenatal care, have an unplanned pregnancy, are young, and have low education [23,24]. Since self-reported gestational age is considered unreliable, gestational age determination was based on ultrasound scanning in the present study.

We found a strong and statistically significant association between exposure to physical violence and PTB and LBW. This finding is in line with other studies [13,15]. In a cohort of 612 Ugandan women attending antenatal care, women who had experienced IPV had a more than three-fold increase in risk of LBW [15]. Likewise, a cohort study from Brazil [13] found that women exposed to any form of violence had a three times increased risk of LBW, although physical violence alone was not associated with a statistically significant increased risk. However, the Brazilian study assessed violence as early as 16 weeks of gestation and might have missed events of physical violence happening after the 16^th^ week. Similarly, misclassification of violence exposure may have influenced the findings in two studies from Mexico and Brazil where no association between IPV and PTB or LBW was found [17,25]. The Mexican study recruited women during their postpartum period and retrospectively assessed exposure to violence during pregnancy, a design which is more likely to have recall bias, and lead to underestimation of prevalence of violence [17]. The Brazilian study recruited women during their antenatal clinic attendance irrespective of the gestational age of their pregnancy and did one to two assessments for exposure to violence during pregnancy [25]. Women who were interviewed before 28 weeks of gestation and reported exposure to violence had only one round of interview while those who didn’t report experiencing violence were scheduled for another interview during their third trimester. However, the exact gestational age at which violence was assessed among those with an appointment for the follow up interview was not documented. If the assessment of violence was done during the early weeks of the third trimester, that may have provided several weeks of un-assessed time up to delivery for possible exposure to violence leading to ultimate misclassification of exposure status to violence.

Several mechanisms are reported for how IPV is associated with PTB and LBW. As an example, in a Tanzanian study, 23% and 38% of all women exposed to physical violence during pregnancy suffered blows to the abdomen [7], which may lead to placental damage, rupture of the membrane and consequently premature uterine contractions [26]. Anxiety and depression, which are often consequences of exposure to violence [6,27], may also lead to behavioral changes in the form of increased tobacco and alcohol consumption and decreased food intake [15]. Such behavioral changes may be associated with inadequate prenatal weight gain which again may result in LBW [28]. In our study, it remains unclear how the women’s exposure to violence was associated with behavioral changes; however, with the exception of alcohol consumption during pregnancy, we found no statistical difference in smoking and BMI between women exposed and not exposed to violence.

This study further showed that women with a history of a previous adverse pregnancy outcome have a higher risk for both IPV and recurrent PTB and LBW than those without such a history. It could be that the previous and current adverse pregnancy outcomes are a result of abuse from the same partner and, through mechanisms explained earlier, result in PTB and LBW. An alternative explanation could be that pregnancies of women who have a history of a previous adverse pregnancy outcome are more at risk [29,30] and thus more likely to end in a repeated adverse outcome if the women are exposed to violence. Exposure to violence may have resulted in anxiety, increased blood pressure (including pre-eclampsia) [31] and depression[6] which may predispose the women to poor health with malnutrition, frequent genital urinary infections and restricted access to medical care due to opposition by the partner [1,7]. These conditions have been shown to be associated with both; exposure to partner violence during pregnancy and recurrent adverse pregnancy outcomes of PTB and LBW [20,32,33].

One of the main strengths of the present study is the high retention rate (98%). Detailed contact information was obtained from all women (that is, address and telephone number) and the research assistants kept contact with the women throughout the study period. The continuous contact created a trustful relationship that enabled the women to report back immediately after delivery to schedule for a post-delivery interview. The use of ultrasound scanning for gestational age determination was another strength of the study. It is recognized that gestational age determination by ultrasound has to be performed before 24 weeks of gestation to be valid; therefore we only included women who were less than 24 weeks pregnant. Women, who are past 24 weeks of gestation when registering for ANC, may have a different risk of both IPV and adverse pregnancy outcomes and our chosen inclusion criteria may have introduced selection bias. However, since the vast majority of the women in the study area do attend ANC before 24 weeks of gestation we believe our findings are representative for women living in the Kilimanjaro Region. Another inherent weakness of the study is the fact that violence is a sensitive topic and cultural norms for disclosure may have led to underreporting. However, a team of female nurses with experience in handling sensitive topics was selected as interviewers; they used an empathetic approach and, as described previously, followed the same women throughout the pregnancy. We trust that this approach enabled us to obtain valid information on violence exposure; however, if underreporting of IPV has occurred, it may have led to an underestimation of the strength of association between IPV and PTB and LBW. Although we adjusted for most recognized risk factors for PTB and LBW, the etiology of PTB and LBW is multi-factorial and we have probably not been able to take all factors into account. Further, the results presented in this study did not control for anxiety and signs of depression. Finally, the study did not include assessment of other forms of violence that women may have been experiencing, such as from household members other than their intimate partner.

## Conclusion

The study highlights IPV as a major public health problem in Tanzania. IPV is common during pregnancy and is linked with maternal suffering and increased risks of PTB and LBW. The risk of PTB/LBW is even stronger if the women in addition to IPV exposure also carry a history of previous adverse pregnancy outcome. The serious consequences of IPV during pregnancy and its high prevalence among pregnant women call for immediate investment in cost-effective interventions addressing IPV. Antenatal care represents an important, and if not used, a missed opportunity to engage in IPV prevention and management. While WHO recommends that violence screening be based on mental and physical health indications associated with violence [34], we recommend that health providers also offer screening for violence to all pregnant women with a history of previous adverse pregnancy outcomes. To provide services for screening, care and treatment of victims exposed to violence; capacity building of staff working in antenatal care coupled with the creation of appropriate settings that enhance disclosure and safety of women victims of IPV is of paramount importance. In addition, strengthening health care facility networks with other stakeholders who support victims of violence is crucial for securing social support for women who are in need and are willing to receive the support.

## Supporting information

S1 File Enrolment interview questionnaire. (DOC)

S2 File Follow up interview questionnaire. (DOC)

S3 File Complete dataset. (XLS)

S4 File Label for variables in the dataset. (XLS)

S1 Table Additional demographic information. (DOC)
