# Supplementary material for: Intimate partner violence during pregnancy and its association with preterm birth and low birth weight in Tanzania: A prospective cohort study
Source: PLoS One. 2017 Feb 24;12(2):e0172540. doi: 10.1371/journal.pone.0172540 (PMC5325295; doi:10.1371/journal.pone.0172540)
Supplement: S1 Table — (DOC) [file pone.0172540.s005.doc]

Table S1: Participants characteristics, prevalence of reporting emotional, sexual or physical violence during pregnancy and the association between the likelihood of IPV (N = 1112, if no other indication).

|  | **Total (%)** | **Among women with IPV^1^ (%)** | **Among women with no IPV (%)** | **p–value^2^** |
| --- | --- | --- | --- | --- |
| *History of Diabetes Mellitus* |  |  |  |  |
| Yes | 6 (0.5) | 4 (66.7) | 2 (33.3) | 0.72 |
| No | 1106 (99.5) | 333 (30.1) | 773 (69.9) |
| *HIV (n=1105)* |  |  |  |  |
| Yes | 48 (4.3) | 25 (52.1) | 23 (47.9) | 0.01 |
| No | 1056 (95.7) | 311 (29.4) | 746 (70.6) |
| *Previous miscarriage (n = 689)* |  |  |  |  |
| Yes | 130 (18.9) | 56 (43.1) | 74 (56.9) | 0.01 |
| No | 559 (81.1) | 166 (37.5) | 393 (70.3) |
| *Previous Still birth (n = 688)* |  |  |  |  |
| Yes | 16 (2.3) | 6(37.5) | 10 (62.5) | 0.60 |
| No | 672 (97.7) | 215 (32.0) | 457 (68.0) |
| *Previous preterm delivery (n = 682)* |  |  |  |  |
| Yes | 22 (3.2) | 11 (50.0) | 11 (50.0) | 0.01 |
| No | 660 (96.8) | 209 (31.7) | 451 (68.3) |
| *Previous LBW delivery (n = 679)* |  |  |  |  |
| Yes | 33 (4.9) | 11 (33.3) | 22 (66.7) | 0.91 |
| No | 646 (95.1) | 208 (32.2) | 438 (67.8) |
| *Wanted to be pregnant now* |  |  |  |  |
| Yes | 845 (76.0) | 247 (29.2) | 598 (70.8) | 0.19 |
| No | 267 (24.0) | 90 (33.7) | 177 (66.3) |
| *Living with partner* |  |  |  |  |
| Yes | 998 (89.7) | 286 (28.7) | 712 (71.3) | <0.01 |
| No | 114 (10.3) | 51 (44.7) | 63 (55.3) |

^1^ Physical, emotional and/or sexual violence

^2^ Results of chi-square test that show statistical difference of exposure to violence
